# Artificial Intelligence in dentistry: Balancing innovation with ethical responsibility

**DOI:** 10.6026/973206300210489

**Published:** 2025-03-31

**Authors:** Navdeep Kaur, Georgy Jacob, Amritbir Singh, Sana Khan, Pragati Dhir, Gandharvi Kakarla

**Affiliations:** 1 Department of Dental surgery, Sri Guru Ram Das Institute of Dental Sciences & Research, Amritsar, Punjab, India; 2 Department of Dental surgery, College of Dental Sciences, Davangere, Karnataka, India; 3 Department of Dental surgery, Babu Banarasi Das College of Dental Sciences, Lucknow, India; 4 Department of Dental surgery, MA Rangoonwala College of Dental Sciences and Research Centre, Pune, Maharashtra, India; 5 Department of Dental surgery, Yamuna Institute of Dental Sciences and Research, Mansurpur, Haryana India; 6 Department of Dental surgery, Dr.NTR University of Health Science, Vijayawada, Andhra Pradesh, India

**Keywords:** Dentistry, ethical implications, artificial intelligence, privacy, security

## Abstract

The transformative impact of Artificial Intelligence on dentistry, focusing on its applications in diagnostic accuracy, personalized treatment planning and clinical efficiency is of interest. The technical aspects of Artificial Intelligence models like convolutional neural networks and artificial neural networks with the ethical principles guiding its integration and the privacy and security challenges involved are discussed. Regulatory frameworks in the United States and Canada are reviewed, highlighting gaps and opportunities for safe Artificial Intelligence adoption. Good Machine Learning Practices and future research directions to mitigate risks are important. Thus, the importance of ethical and legal considerations for responsible Artificial Intelligence integration in dental practice is highlighted.

## Background:

Dentistry is one of the many industries that Artificial Intelligence is changing. Artificial Intelligence refers to the ability of machines to mimic human functions like learning, reasoning and decision-making. In dentistry, Artificial Intelligence is being used to enhance patient care, treatment planning and diagnostic procedures. Artificial Intelligence can also be used to automate certain operations, ultimately improving clinical efficiency and decreasing the human errors [1, 2]. Artificial Intelligence's capacity to evaluate big datasets from multiple sources makes personalized treatment planning possible. Based on a patient's genetic information, radiographs and dental history, the system may suggest customized treatment plans, as well as the long-term success of a treatment strategy. Therefore, Artificial Intelligence is anticipated to play a critical role in augmenting dental technology [1].

## Overview of technical aspects of Artificial Intelligence:

Convolutional neural networks (CNNs) and artificial neural networks (ANNs) are the two artificial Intelligence models most frequently utilized in dentistry. Because these models are made to resemble the neuronal architecture of the brain, artificial Intelligence systems can analyze and interpret massive clinical and imaging data sets with remarkably high accuracy. Convolutional neural networks are specifically utilized for processing and analyzing dental pictures, including X-rays, Computed Tomography scans and 3D images in dentistry, while artificial neural networks are employed more widely for tasks like patient risk assessment and treatment outcome prediction [1, 3 and 4].

## Benefits of artificial intelligence in dentistry:

## Enhanced diagnostic accuracy and treatment planning:

Artificial Intelligence improves diagnostic precision, particularly in areas such as dental radiographs and assists in treatment planning by analyzing patient data and scientific datasets. This leads to better decision-making and outcomes in dentistry [5].

## Personalized treatment plans:

Artificial Intelligence is capable of integrating multi-domain data, including clinical records, medical and dental history, imaging data and systemic conditions. This allows for more customized, individualized treatment plans based on the unique characteristics of each patient [1].

## Continuous monitoring and early detection:

Artificial Intelligence can aid in continuous health monitoring, particularly through wearable devices or other data sources, facilitating early detection of diseases. This is important in preventive dentistry, where timely intervention can prevent the escalation of oral health issues [1].

## Empowering patients:

Technologies make healthcare more participatory by involving patients in their care through self-monitoring, using wearables and apps that provide data for personalized insights and treatment management [1].

## Addressing workforce shortages:

By improving diagnostic accuracy and streamlining processes, Artificial Intelligence can help reduce healthcare costs, particularly crucial as the dental workforce faces shortages [1].

## Facilitating research and discovery:

Artificial Intelligence can assist in research by enabling in silico experimentation and data analysis, which can complement conventional research approaches and help identify new treatment protocols or predictive models for dental conditions [1].

## Increased efficiency:

Artificial Intelligence automates routine tasks, reducing administrative burden and allowing dentists to focus more on patient care. This improves workflow and increases patient satisfaction [6].

## Challenges of artificial intelligence integration in dentistry:

## Integration into clinical practice:

Integrating Artificial Intelligence into routine dental practice remains a challenge, as dental professionals' need proper training to effectively use Artificial Intelligence while maintaining patient-centered care [5, 7].

## Regulatory delays:

The reliance on third-party certifications under the Artificial Intelligence Act (AIA) has led to delays in certification processes, potentially slowing down dental Artificial Intelligence research and innovation [6].

## Education and training of professionals:

The lack of standardized education in Artificial Intelligence for dental professionals poses a significant challenge. While steps have been taken to introduce Artificial Intelligence curricula, more extensive efforts are needed to train professionals in using Artificial Intelligence effectively [6].

## Data quality and accessibility:

Dental data, unlike other medical datasets, is often fragmented, unstructured and of limited size, which can hinder the development of robust Artificial Intelligence models [7, 8].

## Risks associated with artificial intelligence integration in dentistry:

While Artificial Intelligence offers promising potential benefits, several technical and ethical challenges must be carefully evaluated and resolved before Artificial Intelligence systems could be completely incorporated into dental practices. For this paper, these challenges have been categorized into Ethical, Security and Privacy and Legal issues that may arise from concerns with data quality intricacy of the Artificial Intelligence models, compliance with the American Dental Association (ADA) code of conduct and moral dilemmas related with patient data privacy.

## Ethical considerations for artificial intelligence use in dentistry:

American Dental Association Code of Professional Conduct and the Advisory Opinions provide guidance to the dental professionals in order to adhere to high ethical standards of conduct by defining 5 fundamental principles of ethics, namely beneficence, nonmaleficence, autonomy, justice and veracity [9, 10].

The use of technology usually comes with ethical implications that need to be reviewed [11]. The development of both clinical and non-clinical applications of Artificial Intelligence in dentistry thus needs to follow these ethical principles as a guideline, failing which, the use of Artificial Intelligence may raise ethical concerns including but not limited to:

## Non-maleficence & beneficence:

Since the Artificial Intelligence depends on the data sources, there could be risks related to the accuracy of data that may impact the ethics of Beneficence and Maleficence [12]. Dentists can use Artificial Intelligence to augment the diagnosis but should not use it to 'replace' judgement and expertise of licensed professionals [10]. Dental administrative bodies do not currently have an all-inclusive or 'complete structured checklist' to vet Artificial Intelligence systems to minimize all risks associated so that the patient safety is not compromised at any point [13].

## Transparency, informed consent and patient autonomy:

The lack of thorough documentation of datasets, inclusion and exclusion criteria, labeling strategy, training and testing data and the absence of transparency and clear communication with patients to adequately inform about the benefits versus risks or limitations of Artificial Intelligence - based technology can lead to a breach of Transparency, Informed consent and Patient Autonomy [11, 14]. In the absence of adequate information, there is also a risk of patients not having enough autonomy to decide if they are comfortable with the way their data could be used to drive Artificial Intelligence models [15].

## Justice:

Artificial Intelligence systems are trained on large datasets that may contain historical or demographical disparities [14]. If the datasets are unrepresentative and are not critically evaluated and validated to identify or mitigate algorithmic bias, a fair and equitable outcome cannot be expected for all patients [13, 14, 16].

## Equity:

Since Artificial Intelligence Technologies are cost generative, they may increase the existing inequities in healthcare, where the benefits may not be accessible to all individuals equally [11].

## Veracity, trust and accountability:

Even though patients might show increased enthusiasm, dental professionals cannot foster trust and accountability in the absence of clear understanding and explanation to patients on why Artificial Intelligence augmented approach to 'care' is preferred [17]. Currently, it could be difficult to understand how Artificial Intelligence arrives at decision making and can also make it difficult to hold the Artificial Intelligence systems accountable [18]. Absence of ethical guidelines can further raise concerns related to prudence (capacity and expertise), justice, privacy, responsibility, solidarity, autonomy and health care decision making [11, 18].

## Potential impact on dentist- patient relationship:

Another negative impact of Artificial Intelligence overuse in clinical dentistry can be a possible reduction of communication and humanistic care [12, 14]. An increasing popularity of Artificial Intelligence in dentistry and health sciences, therefore, requires a unique and structured ethical model, sometimes called 'Algor-ethics' that ensures the entire lifecycle of an Artificial Intelligence driven process from development to deployment, aligns with the principles of ethics [19]. There is, however, a rare checklist available to reflect 'ethical challenges and demands' of Artificial Intelligence in dentistry [11].

## Privacy and security implications in artificial intelligence-driven dentistry:

The integration of Artificial Intelligence into dentistry brings considerable privacy and security challenges due to the sensitive nature of patient health information. Ensuring data confidentiality, integrity and protection is essential for maintaining patient trust and adhering to regulatory standards.

## Privacy implications:

## Data anonymization and the risk of re-identification:

Anonymizing patient data is essential in Artificial Intelligence applications to protect individual privacy. However, recent studies have shown that even de-identified data can be re-identified, particularly when combined with external datasets. An approach like 'Differential privacy', which introduces statistical noise to the data, can help obscure individual identities while preserving the utility of large datasets for Artificial Intelligence training and is thus required to ensure data anonymization, reducing the risk of re-identification [15, 20].

## Control and ownership of patient data:

As Artificial Intelligence systems in dentistry often operate through public-private partnerships, patients' data may be controlled by commercial entities. This introduces privacy concerns, particularly when for-profit motives might conflict with patient privacy interests. However, to align with ethical and legal requirements such as the Health Insurance Portability and Accountability Act (HIPAA) in the United States of America, General Data Protection Regulation (GDPR) in the European Union (EU) and Personal Information Protection and Electronic Documents Act (PIPEDA) in Canada, governance bodies need to monitor that Artificial Intelligence development companies are compliant to ensure that patients retain ownership and control over their health data, including the right to access, delete, or restrict its use [15, 20, 21 and 22].

## Security implications:

## Data breaches and cyber-security risks:

Artificial Intelligence systems in dentistry are frequently cloud-based, increasing the risk of unauthorized access and data breaches, which can expose sensitive patient information to cyber-attacks. Implementing advanced cyber-security measures, such as data encryption protocols and blockchain technology for secure data management, can enhance protection of patient information. Blockchain's decentralized ledger provides a tamper-resistant storage solution, while homomorphic encryption allows data to be processed without decryption, protecting confidentiality during Artificial Intelligence processing [15].

## Public-private partnerships and data security oversight:

The commercial control of Artificial Intelligence technology in healthcare can lead to data security vulnerabilities, as private entities may prioritize business interests over patient privacy. Regulatory oversight and strong contractual agreements are thus essential to ensure that private custodians prioritize data protection and comply with healthcare data security standards [15].

## Accountability and transparency in artificial intelligence systems:

Establishing accountability in Artificial Intelligence -driven dentistry is essential for maintaining patient trust. Given that Artificial Intelligence models can be complex and operate in "black box" environments, it is crucial to define responsibility-whether with the Artificial Intelligence developer, healthcare provider, or overseeing regulatory body. Transparent Artificial Intelligence models can enable dental professionals to explain how Artificial Intelligence -driven decisions are made, addressing security and ethical concerns and ensuring that any potential issues are promptly identified and managed [23].

## Ethics at the core of privacy and security:

Privacy and security challenges in Artificial Intelligence-driven dentistry are deeply rooted in ethical principles such as autonomy, non-maleficence, beneficence, justice and veracity. Safeguarding patient data is not just a technical requirement but a moral obligation to maintain trust and uphold professional integrity. Robust privacy protections, transparent data practices and advanced security measures ensure that the integration of Artificial Intelligence technologies aligns with ethical standards while prioritizing patient rights. By addressing these concerns thoughtfully, the dental community can foster equitable and trustworthy Artificial Intelligence-driven care, ensuring that innovation remains guided by ethical responsibility [10, 24].

## Legal frameworks for artificial intelligence integration in dentistry:

To Introduce Artificial Intelligence Health products in United States of America (USA) and Canada, best practices and Regulatory processes are maintained by Food and Drug Administration (FDA) Regulation and Health Canada respectively. In Canada, medical devices (including Artificial Intelligence related) are regulated by Health Canada through a licensing system set up under the Food and Drugs Act of 1985 and the Medical Devices Regulations from 1998. Similarly, in USA, Artificial Intelligence health products that qualify as medical devices are regulated and evaluated by Food and Drug Administration for their safety and effectiveness [25].

## Health insurance portability and accountability act (USA), personal information protection and electronic documents act (Canada) compliance:

Artificial Intelligence health products must comply with the Health Insurance Portability and Accountability Act (HIPAA) of 1996, United State of America and Personal Information Protection and Electronic Documents Act, Canada (PIPEDA), ensuring patient data privacy and security. However, Health Insurance Portability and Accountability has certain gaps since it only covers specific information generated by 'covered entities' or their 'business associates' and thus does not cover much of the health-related data collected by agencies like Facebook, Apple, IBM, Google, Amazon *etc.* that invest heavily on the Artificial Intelligence in healthcare, or, user-generated health information, *e.g.*, Facebook posts about health status or disease [25, 26].

## Regulatory process of Artificial Intelligence based healthcare products:

Food and Drug Administration provides guidance and uses Clinical Decision Support (CDS) Software to determine if certain medical software is regulated as a Medical Device. Certain medical softwares can be exempted from being classified as medical devices based on the 21st Century Cures Act. This reduces the regulatory burden. However, if the Artificial Intelligence product qualifies as a "medical device", it must go through the regulatory process to be authorized by Food and Drug Administration [25]. In Canada, Medical devices are classified from class I to IV, in which class I represents lowest risk while class IV devices represent highest risk. Similarly, in the United States devices are classified from I to III, with I being of lowest risk and exempt from premarket notification, class II requiring premarket notification and class III requiring premarket approval [25, 27]. If an Artificial Intelligence product qualifies as a "medical device", it can fall into any of these categories depending on its use and risk level and must follow this licensing process, even if it doesn't come with any physical parts. As per Health Insurance Portability and Accountability and Health Canada, Class I devices do not need a License. Currently, most of the Artificial Intelligence products in the market for dentistry are software or class I devices and thus, do not require licensing procedures [25, 27]. Software pre-cert program allows for streamlined Health Insurance Portability and Accountability review for some low-risk software-based medical devices [25].

Artificial Intelligence products that pose low to moderate risk and fall in Class II require a device-specific license. These could be the Artificial Intelligence systems that require human oversight. Class III devices in United State of America may include Artificial Intelligence devices that pose a low risk, while Class III devices in United State of America and Class IV devices in Canada include high risk Artificial Intelligence devices and require a device-specific license. This has been summarized below in the Table 1 [25, 27].

Food and Drug Administration publishes and periodically updates a list of medical devices that incorporate Artificial Intelligence / Machine Learning, primarily based on 'publicly available information provided in the summary descriptions of their marketing authorization document', that have 'met the Artificial Intelligence's applicable premarket requirements, including a focused review of the devices' overall safety and effectiveness, which includes an evaluation of appropriate study diversity based on the device's intended use and technological characteristics' [28].

In the 2023 published list of 521 authorized Artificial Intelligence-enabled medical devices, none of the devices listed used Generative Artificial Intelligence or large language models. Dental devices made up less than 1% of Food and Drug Administration cleared Artificial Intelligence enabled devices, with no new devices authorized for the panel 'dental' (1 in 2023 vs 1 in 2022) [29]. In the list published for 2024, Food and Drug Administration cleared a large majority of Artificial Intelligence devices (about 97 % of those on the list as of August 2024) through its less rigorous, faster and comparatively cheaper market authorization option for moderate- risk devices [30]. Yet, the list only had 3 Artificial Intelligence devices that were listed under 'Dental' - 2 Orthodontic Softwares and a Dental Navigation System [28]. A large number of devices on both these lists continue to be related to Radiology [28, 29 and 30]. Since many of the ethical issues have legal ramifications which include safety and effectiveness of Artificial Intelligence operations, liability, data protection and privacy, cyber-security and respect of Intellectual property, it is vital to review the legal frameworks and recent developments to identify and address any potential gaps in the current regulatory system [25].

## Good machine learning practice (GMLP):

Ensuring the safety and effectiveness of Artificial Intelligence (AI) is extremely important. Stakeholders can help implement Artificial Intelligence successfully in dentistry by ensuring that the data used is both reliable and specific for the population it is used upon. They should focus on regularly updating the software and being open about their products, including any potential issues like data biases. Additionally, it is crucial to have proper oversight to guarantee the safety and effectiveness of Artificial Intelligence systems [25]. The Food and Drug Administration, Health Canada and the United Kingdom's Medicines and Healthcare products Regulatory Agency (MHRA) have collaborated to establish 10 guiding principles. These are intended to assist in creating Good Machine Learning Practice (GMLP) for Artificial Intelligence technologies used in healthcare [31]. These principles will be a good guide to address the issues of cyber-security, privacy, algorithmic bias and transparency.

## Future challenges and research directions to mitigate risks associated with Artificial Intelligence use in dentistry:

Artificial Intelligence has yet to fully integrate into routine dental practice due to challenges such as limited data availability, lack of methodological rigor and ethical concerns. As Artificial Intelligence evolves, patient acceptance is expected to grow and Artificial Intelligence-driven tools may enhance communication and diagnostic precision without replacing human expertise. However, concerns persist about data privacy, security, accuracy and the risk of depersonalized care [1, 7, 32 and 17].

Hence, ethical and legal standards must be incorporated into Artificial Intelligence development, with transparency and safeguards for data protection [24]. Dental Curricula should integrate Artificial Intelligence into dental education with a focus on ethical and responsible use of Artificial Intelligence, by including training modules on critical thinking and patient communication skills to discuss the effective use of Artificial Intelligence as an adjunct to decision making, as well as the limitations and biases of Artificial Intelligence models and technology [33]. Educating patients about Artificial Intelligence complementary role in dentistry is also essential to build trust. Ethical guidelines, review boards and regular audits should be established to monitor Artificial Intelligence impact and incorporate patient feedback [14, 24 and 34].

## Conclusion:

Artificial intelligence holds great potential to transform dental care, but responsible integration requires addressing ethical, security, privacy and legal considerations to ensure compliance and maintain quality care.

## Figures and Tables

**Table 1:** Risk based classification of medical devices in the USA and Canada

	**Medical Device Classification**	
Risk level	Canada	USA
Low	Class I	Class I
Medium	Class II	Class II
Medium-High	Class III	Class III
Highest	Class IV	
